# Changes of retinal oxygen saturation during treatment of diabetic macular edema with a pre-defined regimen of aflibercept: a prospective study

**DOI:** 10.1007/s00417-021-05319-5

**Published:** 2021-09-01

**Authors:** Somar M. Hasan, Martin Hammer, Daniel Meller

**Affiliations:** Department of Ophthalmology, Jena University Hospital, Am Klinikum 1, 07747 Jena, Germany

**Keywords:** Diabetic retinopathy, Retinal oxygen saturation, Diabetic macular edema, Anti-VEGF therapy, Aflibercept

## Abstract

**Purpose:**

To study the effect of anti-VEGF therapy for diabetic macular edema (DME) on retinal oxygen saturation (O_2_S) and its correlation with functional and anatomical changes of retinal tissue.

**Methods:**

An interventional prospective single group study. Included were 10 eyes of 10 patients with visually significant DME which received a fixed regimen of intravitreal aflibercept every 4 weeks for 5 months, followed by 3 injections every 8 weeks, and were controlled monthly. Visual acuity (VA), central retinal thickness (CRT), arterial (aO_2_S), venous (vO_2_S) and arterio-venous difference (AVdO_2_S) retinal oxygen saturation were noted monthly. Changes after 5th (V6) injection and on last follow-up (V12) were studied. Correlations of different parameters were analyzed.

**Results:**

The aO2S did not change whereas vO_2_S decreased (62.2 ± 9.4 pre-op to 57.2 ± 10.5 on V6, *p* = 0.03). This remained unchanged at 59.4 ± 13.2 on V12 (*p* = 0.2) and was accompanied by an increase of AVdO_2_S (40.8 ± 8.3 pre-op to 44.8 ± 10.6, *p* = 0.03 on V6) which was followed by a non-significant decrease to 41.8 ± 11.3 on V12 (*p* = 0.06). We found no correlation between BCVA and aO_2_S. However, mild correlation between BCVA and both vO_2_S and AVdO_2_S (*r* = −0.2 *p* = 0.035 and *r* = 0.185 *p* = 0.05 respectively) was found. No correlation was found between CRT and aO_2_S, vO_2_S, or AVdO_2_S.

**Conclusions:**

During DME treatment with fixed regimen of intravitreal aflibercept over 11 months, we observed a reduction of vO_2_S and increase of AVdO_2_S which correlated with BCVA but not CRT. This could be explained by increasing consumption of O_2_S in the central retina and, possibly, by re-perfusion process.

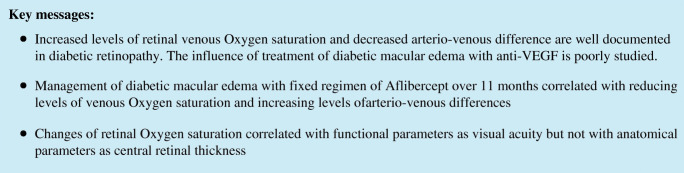

## Introduction

Changes of retinal vessel oxygen saturation (O_2_S) are known in eyes with diabetic retinopathy (DR). Increased arterial (aO_2_S) and venous (vO_2_S) retinal oxygen saturation levels and decreased extraction were measured with increasing severity of DR [1–3]. This could be explained by three mechanisms: (1) capillary nonperfusion and shunting, (2) thickening of capillary vessel walls, and (3) greater affinity of hemoglobin HbA1C for oxygen in diabetic patients [3, 4]. In eyes with capillary shunting, there is bypassing of blood flow of parts of capillary network resulting in non-perfusion and reduced tissue oxygenation. In addition, thickening of capillary walls results in a higher diffusion barrier for oxygen between vessel lumen and tissue [3]. The presence of diabetic macular edema (DME) correlated with more pronounced increase of venous retinal oxygen saturation and decreased extraction compared with the DR without DME [2, 4] and might be explained by reduced retinal autoregulation by increasing severity of diabetic retinopathy [5]. However, a secondary effect of lower oxygen consumption of the central retina as a result of reduced function of the retinal cells cannot be excluded.

The first-line therapy of fovea involving DME is intravitreal injection of vascular endothelial growth factor (VEGF) inhibitors and is known to improve visual acuity and reduce central retinal thickness (CRT). However, it is not known if improvement of visual acuity and reduction of DME correlate with changes of retinal O_2_S. Such correlation might offer new insights which help in understanding the pathophysiology of the disease and its reaction on the anti-VEGF therapy. Treatment of vision threatening DME with 3 monthly injections of intravitreal ranibizumab followed by pro re nata administration did not show significant changes of retinal O_2_S levels in spite of achieving improvement of visual acuity and reduction of macular edema [6]. Similar results were found after 3 monthly injections of intravitreal aflibercept [7]. On the other hand, studies reporting 12-month outcomes of treatment with ranibizumab or aflibercept showed that improvement of central retinal thickness can progress till month 12 accompanied with improvement of visual acuity [8, 9]. The aim of this study was to investigate the progression of retinal O_2_S during a 12-month treatment period of visually significant DME with intravitreal aflibercept using a fixed treatment regimen and to study its correlation with functional (best corrected visual acuity—BCVA) and anatomical (CRT) parameters.

## Methods

This is an interventional prospective single-group non-randomized, non-controlled study. We included patients with non-proliferative diabetic retinopathy and diabetic macular edema involving the fovea and resulting in vision loss. Inclusion and exclusion criteria are listed in Table 1. All patients received a recommended fixed regimen of five intravitreal injections of aflibercept 2 mg/0.05 ml (Eylea, Bayer Vital AG, Leverkusen, Germany) every 4 weeks followed by three injections every 8 weeks. Patients were followed monthly according to the pre-defined protocol as shown in Table 2. Before starting treatment, every patient underwent a thorough ocular examination including BCVA determined by ETDRS standards, Goldmann applanation intraocular tonometry, and slitlamp bio-microscopy for the anterior and posterior segment in mydriasis including staging of the DR using ETDRS grading system. O_2_S was measured and CRT was documented using optical coherence tomography (OCT). All patients underwent fundus fluorescence angiography (FA). These examinations (except for FA) were repeated monthly after the first injection for 11 months.

**Table 1:** Inclusion and exclusion criteria

Inclusion criteria	Patients with non-treated diabetic macular edema
	Able to sign a written informed consent
	Age between 21 and 80 years
	Able to complete the study protocol
	Best corrected visual acuity between 20/400 and 20/25 in the study eye
	Manufacturer’s criteria for aflibercept treatment
Exclusion criteria	Proliferative DR or non-proliferative DR requiring or expected to need laser photocoagulation during the study period.
	Any previous ocular surgical intervention other than non-complicated cataract surgery (this should have been performed 8 weeks before inclusion)
	Any retinal pathology other than DR (vascular occlusion, hereditary retinal dystrophies, vitreomacular traction, etc.)
	Any macular pathology other than DME (any form or stage of macular degeneration, macular hole, macular edema of other reason than DME .. etc.)
	Spherical equivalent of more than +6.0 Diopters or less than −3.0 Diopters
	Systemic diseases which might affect retinal O2S (COPD, renal insufficiency, etc.)
	Hypertension with hypertensive retinopathy ≥ II°
	History of stroke, transient ischemic attack or myocardial infarction
	Status post intravitreal injections
	Media opacities affecting fundus examination
	Any acute infection of the eye
	Advanced glaucoma with a cup to disk excavation ≥0.8
	Pregnancy or lactation

Abbreviations: *DR* diabetic retinopathy

**Table 2:** Study protocol

	V 0	V 1	V 2	V 3	V 4	V 5	V 6	V 7	V 8	V 9	V 10	V 11	V 12
IVIA		X	X	X	X	X		X		X		X	
Visus	X		X	X	X	X	X	X	X	X	X	X	X
IOP	X		X	X	X	X	X	X	X	X	X	X	X
Slitlamp	X		X	X	X	X	X	X	X	X	X	X	X
O_2_S	X		X	X	X	X	X	X	X	X	X	X	X
OCT	X		X	X	X	X	X	X	X	X	X	X	X

Abbreviations: *IVIA* intravitreal injection of aflibercept, *IOP* intraocular pressure, *O*_*2*_*S* retinal oxygen saturation, *OCT* optical coherence tomography

### Measurement of oxygen saturation

The pupil was dilated with tropicamide 5.0 mg/mL (Mydrum eye drops; Bausch + Lomb, Berlin, Germany) as the five photos of the retina were taken with the retinal vascular oximeter (IMEDOS Systems UG, Jena, Germany), focusing on the optic nerve head and the parapapillary vessels with intervals of approximately 30 s between them. Arteries and veins were marked by the same experienced examiner (SMH); and O2S in the arteries, veins, and the arteriovenous difference (AV-D) were automatically measured and averaged over a circumpapillary ring with an inner and outer diameter of 2 and 3 disk radii, respectively. The software VesselMap 3.60, a component of the oximeter, was also used. Optical densities of the vessels were measured as the logarithmic ratio of the fundus reflection at the vessel and besides the vessel. To exclude specular reflex from the vessel, pixels with a reflection above 20% over the mean value were excluded. The ratio of the optical densities at 610 nm to that at the isosbestic wavelength of 548 nm is proportional to the vessel hemoglobin oxygen saturation after compensation for vessel diameter and fundus pigmentation. A linear relationship between the optical density ratio and the relative oxygen saturation measure was established by calibration. Vessel tracking and calculation of the oxygen saturation were done automatically by the software of the device. The reproducibility of the measurement was shown to be 2.5% in arteries and 3.25% in veins (mean standard deviation of repeated measurements) [10]. Examination was performed in a dark room without any illumination source other than the retinal oximeter.

### Optical coherence tomography

As the pupil was still dilated following O2S measurement, OCT examination of the central retina (Cirrus HD-OCT 5000 Carl-Zeiss Meditec, Oberkochen, Germany) with use of the central macular cube settings and help of eye-tracker was performed. Examined central retina was divided using the ETDRS grid and thickness of the central (1 mm) ring was documented. Included were only images with a quality of 5/10 or better.

The study was performed in accordance with the Declaration of Helsinki, and a signed informed consent was obtained from all participants before any study related examination was performed. The study protocol was approved by the ethical commission of the Jena university hospital, Germany.

### Patients

The study enrolled 11 eyes of 11 patients with type-2 diabetes and non-proliferative diabetic retinopathy with diabetic macular edema and foveal involvement resulting in reduced visual acuity. One patient was lost from follow-up after the sixth visit because of hospitalization as a complication of her diabetes so that 10 eyes of 10 patients were available at the last visit for statistical analysis. No patient received anti-VEGF therapy to the other eye during study time.

BCVA, CRT, intraocular pressure, and vascular retinal O_2_S were compared on three time points:

- Before starting treatment (visit 0)
- 4 weeks after the 5th injection corresponding to month 5 (visit 6), where the maximum effect of aflibercept is expected (4 weeks after the 5th injection performed every 4 weeks)
- 4 weeks after the 8th injection corresponding to month 11 (visit 12), where we wanted to measure the effect of a maintenance dose of 3 injections every 8 weeks

Changes of every visit to the visit before were calculated for each of the different parameters (BCVA, CRT, aO_2_S, vO_2_S, and arteriovenous difference oxygen saturation (AVdO_2_S)) through all monthly visits. Correlations of these changes were studied.

### Statistical analysis

The statistical analysis was performed using IBM SPSS Statistics Version 22.0 (IBM Corp., Armonk, NY, USA). All parameters were tested for normal distribution (Shapiro-Wilk test). In normally distributed data, the paired sample *t*-test and Pearson’s correlation test were used. Otherwise, Wilcoxon signed-rank and the Spearman correlation tests were used. All results are presented as mean ± standard deviation. A *p* value of ≤0.05 was considered significant.

## Results

The basic data of all ten patients before starting therapy are listed in Table 3.

**Table 3:** Preoperative data of all 10 eyes of 10 patients

Patient	Sex	Age	Loc	IOP	Stage	CRT	BCVA	aO_2_S	vO_2_S	AVdO_2_S
1	F	62	L	16	Moderate	320	79	100.91	65.284	35.626
2	M	66	R	20	Mild	343	72	100.566	63.614	36.952
3	F	81	L	17	Mild	463	56	92.982	45.404	47.578
4	F	65	L	22	Severe	498	54	100.696	70.162	30.534
5	M	77	L	14	Moderate	270	55	108.922	69.094	39.828
6	M	41	R	21	Mild	384	75	96.342	55.971	40.371
7	F	60	R	18	Mild	390	65	95.602	58.666	36.936
8	M	60	R	14	Severe	494	75	112.638	58.422	54.216
9	M	72	L	13	Severe	395	60	111.628	79.354	32.274
10	M	50	L	16	Moderate	383	51	109.621	55.983	53.638
Mean ± STD		63.4 ± 11.9		17.1 ±3.1	N/A	394 ± 73.99	64.2 ± 10.3	102.99 ± 7.15	62.19 ± 9.44	40.79 ± 8.34

Abbreviations: *LOC* localization, *L* left, *R* right, *IOP* intraocular pressure in mmHg, *Stage* stage of diabetic retinopathy, *CRT* central retinal thickness in μm, *BCVA* best corrected visual acuity in ETDRS letters, *aO*_*2*_*S* arterial retinal oxygen saturation, *vO*_*2*_*S* venous retinal oxygen saturation, *AVdO*_*2*_*S* arteriovenous difference of retinal oxygen saturation

### Changes of BCVA, CRT, IOP, and DR

Changes of these parameters on every visit are listed in Table 4. The BCVA improved on visit 6 from 64.2 ± 10.3 pre-op to 69.8 ± 10.2 ETDRS letters (*p* = 0.05) and remained stable on visit 12 at 71.0 ± 9.3 ETDRS letters (*p* > 0.05). The CRT reduced from 394 ± 73.9 μm pre-op to 308 ± 73.7 on visit 6 (*p* = 0.009) and continued to improve till visit 12 (290.1 ± 64.7 μm, *p* = 0.04). IOP remained stable at 17.1 ± 3.1, 17.2 ± 2.0, and 17.6 ± 2.9 mmHg pre-op, on visit 6 and on visit 12 (*p* = 0.9 and 0.7 respectively). No changes of the stage of DR were observed on visit 12.

**Table 4:** Outcomes over 11 visits (mean ± standard deviation, *p* values are calculated for every visit compared to V0)

Patient	V0	V2	V3	V4	V5	V6	V7	V8	V9	V10	V11	V12
BCVA	64.2 ± 10.3	69.2 ± 9.0	68.8 ± 7.94	69.5 ± 9.16	68.8 ± 10.38	69.8 ± 10.22	69.5 ± 9.70	69.6 ± 8.99	69.2 ± 9.37	69.7 ± 9.74	70.5 ± 9.25	71 ± 9.35
CRT	394 ± 73.98 (*p* = 0.005)	316.4 ± 62.05 (*p* = 0.005)	306.4 ± 66.0 (*p* = 0.005)	301.5 ± 71.75 (*p* = 0.005)	302.7 ± 69.39 (*p* = 0.009)	308 ± 73.71 (*p* = 0.009)	299.2 ± 67.08 (*p* = 0.005)	296.9 ± 62.99 (*p* = 0.007)	295 ± 75.65 (*p* = 0.005)	293.5 ± 67.26 (*p* = 0.005)	295.1 ± 64.08 (*p* = 0.005)	290.1 ± 64.67 (*p* = 0.005)
IOP	17.1 ± 3.1	15.2 ± 3.0	15.7 ± 3.5	15.94 ± 2.7	16.5 ± 3.3	17.17 ± 2.0	16.7 ± 2.7	16.4 ± 2.4	17.8 ± 2.5	16.8 ± 1.3	17.4±.1.3	17.6 ± 2.9
aO_2_S	103.0 ± 7.1	102.2 ± 8.3 (*p* = 0.3)	104.8 ± 7.4 (*p* = 0.1)	103.8 ± 8.4 (*p* = 0.2)	103.5 ± 8.2 (*p* = 0.6)	102.0 ± 7.3 (*p* = 0.4)	102.0 ± 8.1 (*p* = 0.5)	101.2 ± 7.0 (*p* = 0.2)	100.2 ± 8.5 (*p* = 0.1)	100.9 ± 8.5 (*p* = 0.07)	101.3 ± 7.4 (*p* = 0.3)	101.2 ± 7.6 (*p* = 0.08)
vO_2_S	62.2 ± 9.4	62.0 ± 11.1 (*p* = 0.9)	64.6 ± 9.6 (*p* = 0.2)	60.4 ± 9.8 (*p* = 0.4)	62.9 ± 11.2 (*p* = 0.6)	57.2 ± 10.5 (*p* = 0.04)	62.9 ± 9.7 (*p* = 0.9)	58.1 ± 10.5 (*p* = 0.07)	58.1 ± 11.0 (*p* = 0.2)	59.3 ± 10.8 (*p* = 0.07)	59.6 ± 11.3 (*p* = 0.5)	59.4 ± 13.2 (*p* = 0.2)
AVDO_2_S	40.8 ± 8.3	41.1 ± 8.4 (*p* = 0.6)	40.2 ± 9.6 (*p* = 0.6)	44.7 ± 11.4 (*p* = 0.1)	40.6 ± 11.2 (*p* = 0.5)	44.8 ± 10.6 (*p* = 0.037)	41.1 ± 10.6 (*p* = 0.5)	43.0 ± 10.8 (*p* = 0.1)	39.1 ± 9.1 (*p* = 0.8)	41.7 ± 9.5 (*p* = 0.5)	38.8 ± 9.0 (*p* = 0.1)	41.8 ± 11.3 (*p* = 0.06)

Abbreviations: *V* visit, *BCVA* best corrected visual acuity in ETDRS letters, *CRT* central retinal thickness in μm, *IOP* intraocular pressure in mmHg

### Changes of the retinal O_2_S

**Fig. 1:**
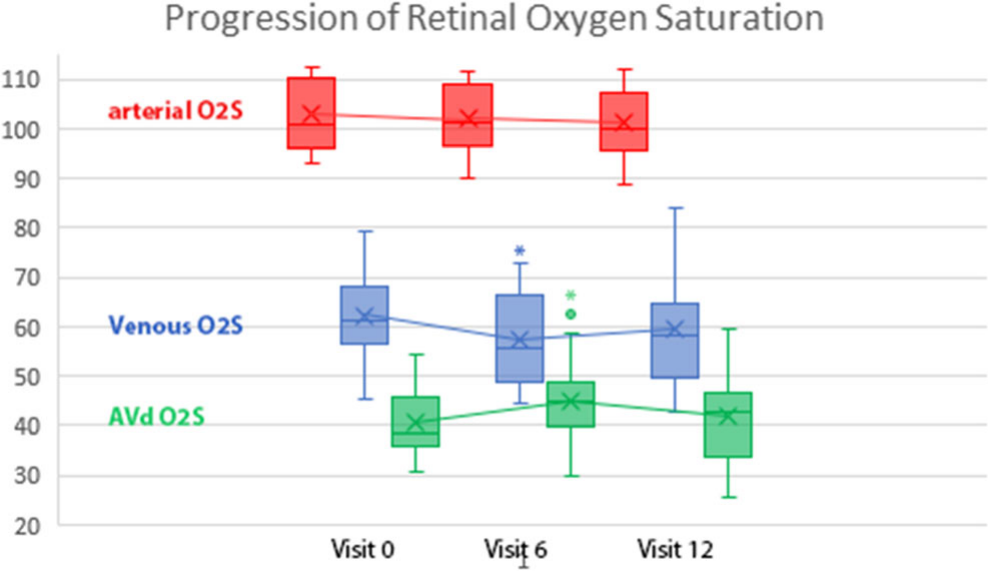
Progression of retinal oxygen saturation on visit 0, visit 6, and visit 12. Graphic generated using IBM SPSS Statistics Version 22.0 (IBM Corp., Armonk, NY, USA). The * sign points to statistically significant difference

The aO_2_S remained unchanged (pre-op 103.0 ± 7.1 to 102.0 ± 7.3 on visit 6 and to 101.2 ± 7.6 on visit 12, *p* = 0.39 and 0.74 respectively), although showing a downward trend. We observed a significant reduction of vO_2_S from 62.2 ± 9.4 pre-op to 57.2 ± 10.5 on visit 6 (*p* = 0.03) which then remained unchanged at 59.4 ± 13.2 on visit 12 (*p* = 0.2). This was accompanied by an increase of AVdO_2_S saturation from 40.8 ± 8.3 pre-op to 44.8 ± 10.6 (*p* = 0.03) on visit 6 and followed by a non-significant decrease to 41.8 ± 11.3 on last visit (*p* = 0.06) (Fig. 1).

Studying the correlations of changes between the different parameters (change of every visit to the visit before) showed a significant correlation of BCVA with CRT (*r* = −0.32, *p* > 0.001, Pearson correlation test). We found no correlation between BCVA and the aO_2_S (*p* > 0.05). However, we found a mild but significant correlation between the BCVA and both the vO_2_S and the AVdO_2_S (*r* = −0.2, *p* = 0.035 and *r* = 0.185, *p* = 0.05 respectively). No correlation was found between the CRT and the aO_2_S, vO_2_S, or AVdO_2_S.

## Discussion

Different pathologic features are associated with DR including apoptosis of vascular endothelial cells and pericytes, thickening of basement membrane, capillary occlusion, and break down of the blood-retinal barrier (BRB) [11]. Synthesis of VEGF is upregulated by different mechanisms including tissue hypoxia [12], and its concentrations in diabetic patients are 5 times that of age-matched controls [13]. Treatment of visually significant DME with anti-VEGF is now a standard of care and leads to improvement of VA and reduction of CRT. The effect of anti-VEGF therapy on the retinal oxygen saturation and its correlation with the functional and anatomical changes during therapy is not well studied. In our study, we report for the first time the changes of retinal O_2_S during treatment of DME with a pre-defined treatment regimen over a 12-month period.

We observed an improvement of the visual acuity from 64.2 ± 10.34 letters pre-op to 69.5 ± 9.7 on visit 6 and to 71.0 ± 9.36 on the last visit. Similar results were found in other studies using aflibercept and giving a mean of 8 injections in 1 year [14]. In other studies, an even further improvement was achieved when using a different treatment protocol which allowed laser photocoagulation and more injections [9]. The central retinal thickness reduced from 394 ± 73.9 μm pre-op to 308 ± 73.7 after on visit 6 (*p* = 0.009) and continued to reduce till last visit to 290.1 ± 64.7 (−104 μm on month 12). This is also in accordance with published data [14].

Studying progress of retinal O_2_S, we did not see significant changes of aO_2_S during treatment course but we observed a significant reduction of vO_2_S, a change of −8% which remained stable on last visit and was accompanied by an increase of AVdO_2_S on visit 6 and remained stable on last visit. This contradicts results of other studies, where a- and vO_2_S did not change during treatment with aflibercept or ranibizumab. These studies, however, had either a shorter follow-up time of 3 months [7], which might not be sufficient in many cases to control the disease and reach the end results functionally or anatomically or used another treatment regimen like a loading dose of 3 injections followed by pro re nata schema and allowed laser photocoagulation after month 3, resulting in less injections (6.6 ± 2.5) in 1 year. However, a higher central retinal thickness was found after 6 months (346.2 ± 109.4 μm) compared to our study [6]. The effect of laser photocoagulation on retinal O_2_S is controversial. This is why patients who might need laser photocoagulation during the study time were excluded in our study [1, 15, 16].

The decrease of vO_2_S and increase of AVdO_2_S could be explained by different mechanisms. (1) Improvement of severity of DR following treatment with anti-VEGF with aflibercept associating with more improvement at year 1 and 2 even in the absence of re-perfusions might have played a role [17, 18], as less severe retinopathy is associated with lower vO_2_S and higher AVdO_2_S [1, 2]. In our study, we did not observe an improvement of the DR but we had 7 of 10 eyes (70%) with a mild to moderate disease where improvement might not be detected through the ETDRS scale. Eyes with active proliferative DR were excluded from study. (2) A re-perfusion process in areas of non-perfusion after treatment of DR with anti-VEGF [19] was reported, although this is controversial [18]. The reperfusion may result in increased consumption of O_2_ by the re-perfused retinal areas and reduction of the vO_2_S. These changes of vO_2_S were not accompanied with changes of aO_2_S but with an increased AVdO_2_S, which might point to the possibility of increasing oxygen consumption of the retinal tissue during treatment with aflibercept. In mice retinas suffering from oxygen-induced injury and developing hypoxia, Arias et al. reported a significant improvement of the function of amacrine cells measured with electroretinogram after treatment with aflibercept compared with non-treated mice [20]. These changes were accompanied with improvement of the a and b waves pointing to an improved retinal function. Improvement of inner retinal cells, especially amacrine cells, might have led to increased O_2_ consumption and might partly explain our results.

The vO_2_S correlated mildly but significantly with the visual acuity. This inverse correlation, where increased visual acuity was associated with lower vO_2_S levels, could also be explained by increasing O_2_ consumption as retinal cells restore their function achieving a better BCVA, independent of the central retinal thickness and the anatomical progression of the central retina. It is well known that oxygen consumption is higher in the parafoveal retina compared with the peripheral retina and it even increases in case of hypoxia which is a main component of the pathophysiology of DME [21]. As to our knowledge, our study is the first to report the correlations of the O_2_S with functional (BCVA) and anatomical (CRT) parameters and to show a significant correlation of the changing vO_2_S with the visual acuity.

Our study has some limitations: we had a small sample of 10 eyes and studies with larger samples are needed to verify our results. Wide field fluorescein angiography or OCT-angiography was not part of our study so that we were not able to evaluate the role of re-perfusion or quantitatively grade the severity of DR in our sample although the clinical grading system which we used showed no changes of the severity of DR during study period. Still, this system does not include assessment of peripheral ischemia and its possible contribution to the changes of O_2_S.

## Conclusion

Our study is the first to report the effect of treatment of eyes with DME with fixed treatment regimen of intravitreal aflibercept on retinal vascular O_2_S over a 12-month period and to describe its correlation with BCVA and CRT. Reduction of vO_2_S and increase of AVdO_2_S over the study period and its correlation with BCVA but not CRT could be explained by increased consumption of O_2_S in the central retina and, possibly, by a re-perfusion process. Studies with larger sample size and inclusion of wide field angiography and observing changes of DR could offer a verification and additional explanation of our findings.

## Data Availability

All collected data are available on demand.
